# Delivery of an Incidental Appendiceal Mucinous Neoplasm

**DOI:** 10.7759/cureus.26214

**Published:** 2022-06-22

**Authors:** Madison Bowles, Jessica Y Ng, Hajir Nabi

**Affiliations:** 1 Colorectal Surgery, Department of General Surgery, Logan Hospital, Brisbane, AUS; 2 Surgery, Gold Coast University Hospital, Queensland, AUS; 3 Colorectal Surgery, Logan Hospital, Queensland, AUS

**Keywords:** low-grade appendiceal mucinous neoplasm, cesarean, pregnancy, cytoreductive surgery, pseudomyxoma peritonei, lamn, appendix

## Abstract

Low-grade appendiceal mucinous neoplasms (LAMNs) are rare and non-invasive tumors of the appendix, and their unexpected discovery during surgery can pose challenges to management. To date, only two cases pertaining to LAMNs without peritoneal spread in pregnancy exist in the literature. Here, we present a literature review of appendiceal mucinous neoplasms and discuss our management and operative approach to a large, incidental appendiceal mucinous neoplasm discovered during an emergency cesarean section of a 38-year-old female.

## Introduction

Appendiceal mucinous lesions are characterized by cystic dilatation of the appendix secondary to the accumulation of gelatinous material. Low-grade appendiceal mucinous neoplasms (LAMNs) are rare and non-invasive tumors of the appendix, representing 0.2-0.7% of all appendix specimens. They comprise a group of neoplasms with remarkable histopathologic diversity ranging from simple retention cysts to malignant mucinous adenocarcinoma, with the potential for rupture resulting in the dreaded complication of pseudomyxoma peritonei (PMP). The American Joint Committee on Cancer (AJCC) 8th Cancer Staging Manual defines LAMN as a mucinous neoplasm with low-grade cytology associated with the obliteration of the muscular mucosae, without overt features of invasion. The lamina propriety is frequently effaced, mucosal lymphoid tissues are decreased or absent, and pushing invasion is seen [1].

This case discusses a 38-year-old female with an appendiceal mucinous neoplasm discovered during an emergency cesarean section.

## Case presentation

A 40 + six-week gestation, 38-year-old with an unremarkable medical history and no family history of bowel disease was admitted to the obstetric unit for induction of labor. She had a known cystic structure incidentally found on dating and nuchal ultrasounds. The 10.2 × 3.6 × 5.2 cm cystic structure in the maternal right iliac fossa was of uncertain etiology and separate to the right ovary.

**Figure 1:**
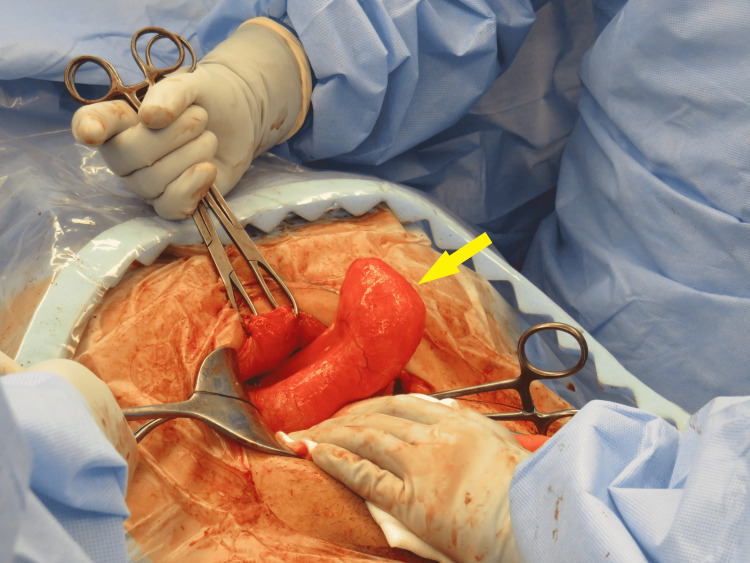
Intraoperative image demonstrating incidental appendiceal mass.

She underwent an emergency cesarean section due to fetal bradycardia secondary to chorioamnionitis and obstructed labor. Following successful cesarean section and delivery of the baby through a Pfannenstiel incision, an inspection of the abdomen was performed to locate the cyst previously identified on ultrasound. An intraoperative general surgical consult was requested due to findings of a grossly distended blind-ending structure in the right lower quadrant (Figure 1). On inspection, a pathologic-appearing, dilated, and fluid-filled appendix was noted but no extra-appendiceal mucin was seen. An Alexis O Wound Retractor (Applied Medical) was inserted to enable adequate access and visualization, the specimen was medialized, and peritoneal attachments were taken down with diathermy. The appendiceal artery was clipped and cut. A stapled cecectomy with a CONTOUR® Curved Cutter Stapler (Ethicon) was performed and the base was oversewn. The patient made an uneventful recovery and was discharged on day three postoperatively.

**Figure 2:**
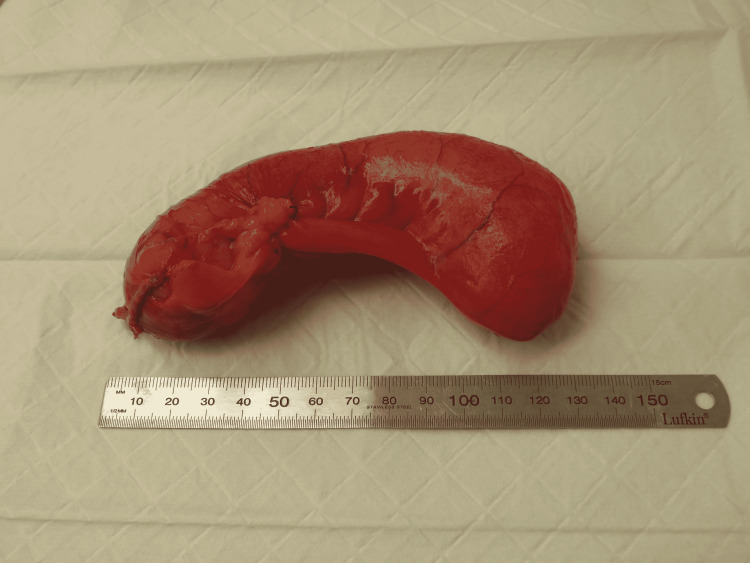
Intact removed appendiceal specimen.

Histopathology revealed a 120 mm × 40 mm appendix. The serosa was injected, and the external surface remained intact. The specimen was fluctuant and cyst-like in texture (Figure 2). The lumen was expanded and contained copious amounts of thick viscous, creamy, mucinous material. Microscopically, the lesion consisted of villiform projections of columnar mucinous epithelium and serrated crypts with closely apposed fibrous tissue. The nuclear atypia was mild. The lesion was confined to the submucosa with no extracellular mucin or atypical epithelium on the serosal surface. The findings were consistent with an LAMN confined to the appendix. There was extracellular mucin with inflammatory cells, but no atypical epithelium was present at the proximal margin. Subsequently, a computed tomography scan of the chest, abdomen, and pelvis was performed (showing no obvious nodal involvement or widespread metastatic disease) in addition to an outpatient colonoscopy which revealed a 40 mm staple line in the cecum and healthy-appearing scar tissue. The patient was offered a right hemicolectomy due to the presence of extracellular mucin with inflammatory cells present at the proximal margin. This was undertaken laparoscopically, and no mucinous neoplasm was seen on histopathology. Additionally, histology revealed no evidence of malignancy, free mucin, or cellular mucin. Twenty-three reactive lymph nodes were identified, and no evidence of metastatic mucinous neoplasm was present.

## Discussion

LAMNs are rare, non-invasive epithelial proliferations that exhibit an absence of infiltrative growth and account for approximately 1% of gastrointestinal neoplasms [1,2]. Histologically, their hallmark feature is a villous or flat proliferation of mucinous epithelium with low-grade atypia [2]. Despite having a relatively good prognosis, their most concerning complication, PMP, is secondary to seeding of mucin into the adjacent peritoneum or rupture during surgical excision and is associated with a high mortality rate [2]. They can be found incidentally, either radiologically or surgically, or can present with non-specific symptoms (abdominal pain, a palpable mass, weight loss) such as appendicitis, intestinal obstruction, or intussusception [2-4]. They most commonly occur in those in the fifth or sixth decades of life and have a slight female preponderance, with ratios of 1:1.4 to 1:1.75 reported in the literature [2,3].

Due to the differing degrees of appendiceal wall fibrosis found in LAMN, the typical colorectal TNM staging is unsuitable [1,4]. pTis (in LAMNs) includes stages pTis, pT1, and pT2 of colorectal cancer. pTis can be further stratified into either pTism, in which fibrosis is confined to the mucosa with cellular mucinous or mucinous epithelium in the inner side of the fibrotic mucosa, or pTisf, in which fibrosis extends beyond the mucosa, involving the muscular propria or the whole layer, with acellular mucinous or mucinous epithelium in the inner side of the fibrotic wall. T3 represents LAMNs showing either acellular mucin or mucinous epithelium involvement of subserosa or mesoappendix, and T4 includes subcategories T4a (penetration of the serosa) and T4b (directly invading adjacent organs or structures) [1,4].

Elevated tumor markers including carcinoembryonic antigen, cancer antigen-125, and carbohydrate antigen-19-9 may be detected in 56.1-67.1% of patients with LAMNs and can be utilized for the surveillance of peritoneal malignancy following treatment [2].

At present, no clear treatment guidelines exist for the management of LAMNs, with the strategies for the surgical approach, adjuvant therapy, and follow-up duration all remaining somewhat controversial [2-5]. Surgically, a lack of consensus remains regarding the required extent of surgical resection and whether laparoscopic versus an open procedure is recommended, with some concerns regarding the increased risk of rupture with a laparoscopic approach and subsequent peritoneal dissemination of neoplastic cells [5]. The most widely utilized approach is an appendicectomy alone, with a right hemicolectomy reserved for cases with infiltration of malignancy into the submucosa, positive appendicectomy margin, or the presence of lymph node metastasis [2-4]. For cases with PMP, more aggressive treatment is usually recommended including resection followed by cytoreductive surgery (CRS) and hyperthermic intraperitoneal chemotherapy (HIPEC) [2-4].

In pregnant patients, appendiceal mucinous neoplasms can be diagnosed on routine abdominal ultrasound. When recognized, abdominal diffusion-weighted magnetic resonance imaging should be ordered for further characterization and to allow for the detection of pools of mucin [6].

Prognosis is determined by the tumor stage, peritoneal mucin spillage, and the presence or absence of PMP [5]. Literature suggests a significant prognostic difference between acellular and cellular mucin, and, therefore, CRS is advocated for relatively early lesions with localized cellular mucin spillage due to the high likelihood of progression to extensive intra-abdominal disease if such cases are treated with only appendicectomy or right hemicolectomy [7].

## Conclusions

LAMNs are rare in clinical practice and require appropriate surgical resection with adequate margins and a wide mesoappendix to allow for the analysis of lymph nodes. Although uncommon, clinicians should maintain a degree of suspicion and consider a differential diagnosis of LAMN upon encountering incidental cystic structures on imaging as further workup may prevent inadvertent events during surgery. Cautious handling and resection of the mucinous neoplasm is crucial (to prevent spillage and obtain an appropriate margin) and may pose a challenge in unexpected circumstances (such as during a cesarean section); hence, generous surgical exposure with appropriate retractors and careful selection of resection methods and instruments is vital in achieving an optimal outcome.
